# What is orgasm? A model of sexual trance and climax via rhythmic entrainment

**DOI:** 10.3402/snp.v6.31763

**Published:** 2016-10-25

**Authors:** Adam Safron

**Affiliations:** Department of Psychology, Northwestern University, Evanston, IL, USA

**Keywords:** rhythms, sex, neural entrainment, trance, orgasm, evolution, mate choice

## Abstract

Orgasm is one of the most intense pleasures attainable to an organism, yet its underlying mechanisms remain poorly understood. On the basis of existing literatures, this article introduces a novel mechanistic model of sexual stimulation and orgasm. In doing so, it characterizes the neurophenomenology of sexual trance and climax, describes parallels in dynamics between orgasms and seizures, speculates on possible evolutionary origins of sex differences in orgasmic responding, and proposes avenues for future experimentation. Here, a model is introduced wherein sexual stimulation induces entrainment of coupling mechanical and neuronal oscillatory systems, thus creating synchronized functional networks within which multiple positive feedback processes intersect synergistically to contribute to sexual experience. These processes generate states of deepening sensory absorption and trance, potentially culminating in climax if critical thresholds are surpassed. The centrality of rhythmic stimulation (and its modulation by salience) for surpassing these thresholds suggests ways in which differential orgasmic responding between individuals—or with different partners—may serve as a mechanism for ensuring adaptive mate choice. Because the production of rhythmic stimulation combines honest indicators of fitness with cues relating to potential for investment, differential orgasmic response may serve to influence the probability of continued sexual encounters with specific mates.

Given that reproduction is the bottom line of evolutionary fitness, it is unsurprising that orgasm would be a source of intense pleasure (Pfaus et al., 2012; Tenk, Wilson, Zhang, Pitchers, & Coolen, 2009; Toates, 2014; Wunsch, 2010). The reinforcing properties and underlying mechanisms of sexual climax have been extensively studied in non-human animals (Coria-Avila, 2012; Giuliano & Clément, 2005; Holstege & Huynh, 2011; Johnson, 2006). Although these mechanisms exhibit extensive homology across species, there may also be important variability based on differences in niche and nervous system complexity (Georgiadis, 2012). Despite several intriguing neuroimaging investigations (Georgiadis et al., 2006; Georgiadis, Reinders, Paans, Renken, & Kortekaas, 2009; Georgiadis, Reinders, Van der Graaf, Paans, & Kortekaas, 2007; Holstege et al., 2003; Komisaruk et al., 2004; Komisaruk & Whipple, 2005), the neurophysiological basis of orgasm remains poorly understood. Here, I introduce a model in which the rhythmic nature of sexual activity is central for understanding the phenomenology of sexual trance and orgasm. Further, I suggest this mechanism implies particular ways in which aspects of sexual responding have been shaped by evolution to promote adaptive mate choice.

## Sexual rhythms and evolution

Orgasm typically – but not always – results from rhythmic stimulation of body parts with high concentrations of sensory receptors (Komisaruk & Whipple, 2011). This stimulation is typically achieved with either physical manipulation of the genitals from body–body contact, or sometimes from vibrotactile mechanical devices. Although orgasm-producing activities are usually focused on the genitals, orgasm is sometimes achieved by stimulating other body parts, and sometimes even through thought alone.^1^

Some aspects of orgasmic experience may be unique to humans, but many of these mechanisms are shared across all mammals, all vertebrates, and even invertebrates (Izhikevich, 2010). Homologous neural structures in hypothalamic, brainstem, and spinal nuclei control the glandular and smooth muscle tissue for ejaculation across all male vertebrates (Truitt & Coolen, 2002). Sexual climax, however, involves more than just the release of male-specific fixed action patterns for ejaculation. Rather, behavioral and physiological processes suggestive of orgasm have been observed in a wide variety of both male and female organisms, involving distinct affective displays (e.g. vocalizations and facial expressions), as well as behavioral reinforcement (Goldfoot, Westerborg-van Loon, Groeneveld, & Slob, 1980; Pfaus et al., 2012; Puts, Dawood, & Welling, 2012; Young & Alexander, 2012).

Although particular details are likely to show interspecies diversity, the ability of males to produce rhythmic stimulation likely has a common adaptive significance as an honest indicator of evolutionary fitness (Broek & Todd, 2009; Darwin, 1872). That is, if it is the case that males vary in their rhythm-producing abilities, and if this variability correlates with genetic quality, then females could select better genes for their offspring if they bias reproduction toward these fitter males. For most of evolution, it may have been the case that rhythmic ability and rhythm sensitivity were primarily selected for in the context of female mate choice. This is not to downplay the importance of male mate choice (Louâpre, Fauvergue, van Baaren, & Martel, 2015), and for reasons that will be discussed below, it may have been the case that rhythmic virtuosity was important for both male and female humans. Indeed, dance is often part of courtship and can function as a pre-copulatory mate selection mechanism in and of itself (Broek & Todd, 2009; Hugill, Fink, & Neave, 2010; Neave et al., 2010; Röder, Weege, Carbon, Shackelford, & Fink, 2015).

The possible adaptive significance of rhythmic ability is suggested by the complexity of dynamics involved in sexual activities. First, in order to generate steady rhythmic motion, it is necessary for the nervous system to drive motoric effector systems with precise frequencies and amplitudes such that forces spatially and temporally align to regulate the frequency of ongoing oscillations. Establishing rhythms is no small task (Burger, Thompson, Luck, Saarikallio, & Toiviainen, 2014; Merchant, Grahn, Trainor, Rohrmeier, & Fitch, 2015; Merchant & Honing, 2014), even with respect to moving a single body. These control challenges are further compounded when this body is being used to precisely stimulate a separate mechanical system, which may itself be oscillating or gyrating.

Furthermore, optimal sexual interaction involves modulating activity based on cues ranging from gross movements, to vocal signals, to subtle patterns of emotional expression. The more variables to be integrated, the more difficult this integration becomes, and the more sophistication required for the controlling nervous system. Some of these factors would be important for all reproducing organisms; others would be unique to humans. Human sexual performance depends on being capable of not only switching between multiple rhythms, but of inferring the best times for these changes. In this way, sexual interactions may test not only the sensorimotor quality of mates but also the sensitivity of their social intelligence.

Given the challenges described above, the ability to generate precise and flexible rhythmic patterns – and to do so over an extended period of time – could function as an honest indicator of organismic quality (Broek & Todd, 2009; Hugill et al., 2010; Neave et al., 2010; Röder et al., 2015).^2^ Alternatively, this very precision may to some extent be a function of attentiveness, which could indicate overall interest and propensity toward investment (Campbell, 1972; Woodward & Richards, 2005). That is, the more attentiveness devoted to a sexual interaction, the more likely that either the experience or sexual partner is valued, and so the more likely that there will be a desire for future interaction, and so the more likely that there either is or will be investment in that particular relationship. Investment signaling might be a particularly important factor in humans, both because of the high levels of resources necessary for successful child rearing (Geary, 2015) and also because humans can consciously modulate the quality of interactions based on complex social goals (Tomasello, 2014).

Rhythmic ability is fundamental in sexual selection (Darwin, 1872; Fusani, Barske, Day, Fuxjager, & Schlinger, 2014), with similar evolutionary logic applying to human coitus, bird song, and even the courtship of invertebrate insects (Griffith & Ejima, 2009). However, there may be unique mechanisms by which sexual compatibility and orgasm function as tests of mate quality in humans. In many animals, sexual climax may simply be a matter of triggering a switch in operating modes for specific pattern generating nuclei that originally evolved to control ejaculation in males (Truitt & Coolen, 2002). Although such evolutionarily ancient adaptations could be sufficient for explaining many aspects of orgasm, rhythms may also explain important response properties in more recently evolved structures, such as the neo-cortex. Specifically, I propose that rhythms may be particularly likely to affect cortical dynamics via entrainment of neural synchrony, thus enhancing perceptual vividness and emotional intensity.

There have been numerous studies exploring the potential adaptive significance of orgasm. One of the novel contributions of this paper is a characterization of proximate-level mechanistic details by which ultimate-level evolutionary selective pressures were served (Tinbergen, 1963). More specifically, the model presented here can be thought of as a particular adaptive mechanism consistent with the ‘sire choice’ hypothesis described by Puts et al. (2012) in their extensive review of the literature on the evolution of female orgasm in human and non-human primates. The model is also compatible with several other evolutionary hypotheses of orgasm (e.g. facilitating the formation of pair-bonds), although it is likely incompatible with the hypothesis that orgasm is an evolutionarily neutral byproduct in women that only exists by virtue of its positive selection in men (Lloyd, 2006). Rather, the neurophysiological underpinnings of sexual trance and orgasm suggest a mechanism so powerful that it is likely to have had a profound impact on mate choice in both sexes.

## Synchrony, entrainment, and attention

Although a full characterization of neural rhythms is beyond the scope of this article, here I will describe potential means by which enhanced synchronous activity – both *within* and possibly *between* nervous systems (Hasson, Ghazanfar, Galantucci, Garrod, & Keysers, 2012; Hennig, 2014; Sänger, Müller, & Lindenberger, 2012) – may impact sexual experience and functioning. I propose that synchrony promotes the intensity of sexual experience through at least three mechanisms: 1) enhanced summation of excitatory neural activity, 2) increased attention via integration of mutually informing data streams, and 3) maximal driving of neural systems for reward and somatic response.

The brain exhibits rhythmic oscillations at a variety of frequencies (Buzsáki & Watson, 2012), the source of which is the synchronous activity of neuronal populations. On the level of basic neurophysiology, neurons are more likely to fire action potentials if their inputs arrive within a narrow window of time relative to each other (Schutter, 2004). This temporal summation suggests a straightforward role for synchrony in enhancing neural signaling: synchronized neural systems allow inputs to arrive within sufficiently narrow windows of time such that neurons are more likely to transmit further signals by firing.^3^

In terms of cognitive and affective functioning, synchrony likely promotes enhanced coordination of different kinds of information (Deco & Kringelbach, 2016). Experiences have tactile, visual, auditory, olfactory, and gustatory aspects, all of which are associated in specific ways based on their common causation by particular multi-aspect properties of the world (Hayek, 1952). This challenge of bringing together multiple causes into a coherent flow of experience is sometimes called the ‘binding problem’ (Singer, 2001), and synchrony may be crucial for understanding how different aspects of percepts are bound together into coherent wholes (Baars, 2005; Edelman, 2004; Melloni et al., 2007; Tononi, 2008). Synchronous rhythms have been proposed as a basic mechanism of perceptual stability and attentional control, and theoretically, the conditions that promote neural synchrony could enhance the vividness of awareness (Buzsáki & Watson, 2012; Canolty & Knight, 2010; Lakatos, Karmos, Mehta, Ulbert, & Schroeder, 2008; Lutz, Greischar, Rawlings, Ricard, & Davidson, 2004).

Entrainment is a means of enhancing particular synchronous patterns, occurring when a system is influenced to oscillate at a given frequency based on rhythmic stimulation at similar or related frequencies (Canolty & Knight, 2010; Thut et al., 2011). Although still awaiting empirical verification, there are several reasons to believe that sexual rhythms are likely to entrain synchronous brain oscillations.

First, it is clear that at least some degree of rhythmic stimulation facilitates sexual enjoyment (Joannides, 2000; Kaplan, 2013) and that an overly irregular or erratic rhythm can be disruptive. Although an overly predictable pattern can lead to desensitization and habituation, excessive predictability can be circumvented through either introducing novel stimulation, by switching between rhythmic modes, or by increasing rhythmic complexity (Vuust & Witek, 2014; Witek, Clarke, Wallentin, Kringelbach, & Vuust, 2014). The presence of these semi-stable oscillations during sexual activity provides oscillating sensory inputs, thus providing a possible means of entraining neural systems (Teplan, Krakovská, & Štolc, 2011).

A second reason to think sexual rhythms can entrain brain oscillations is their multi-modal richness. Sexual activity (either interpersonal or solitary) frequently involves synchronized rhythmic production of related somatic, visceral, visual, auditory, olfactory, or gustatory signals. The synchronous information from these various sensory modalities provides multiple potential channels for entrainment of an individual nervous system, with potential for interpersonal entrainment if multiple nervous systems are simultaneously entrained to the same source of rhythmic stimulation (Bachrach, Fontbonne, Joufflineau, & Ulloa, 2015). Furthermore, the generation of rhythmic sexual actions represents an additional converging data stream (Brown, Friston, & Bestmann, 2011) and potentially powerful entraining signal. Moreover, the brain combines information from these multiple sensory streams such that one modality is capable of resolving ambiguity present in another (McGurk & MacDonald, 1976; Tiippana, 2014). This multimodal informational synergy may not only provide clearer consciousness of percepts relating to sexual stimulation but may also provide synergistically greater entrainment effects. Indeed, increased comprehension of stimulus characteristics has been observed to increase neural entrainment from the rhythmic patterns associated with language and music (Burger et al., 2014; Doelling & Poeppel, 2015; Nozaradan, Peretz, & Mouraux, 2012; Obleser, Herrmann, & Henry, 2012; Peelle, Gross, & Davis, 2013). Multimodal synergy may serve similar functions for enhancing the potency of sexual stimulation (Rowe, 1999).

Further, sexual stimulation is such that greater attention is likely to result in greater enjoyment. This is evidenced by introspection as well as best practices from sex therapy (Brotto, Basson, & Luria, 2008; Prause, Janssen, & Hetrick, 2008). The more one can attend, the better sex feels; and the better sex feels, the more one wants to attend. This setup provides a bidirectional and mutually reinforcing relationship between pleasure and attention, thus instantiating positive feedback toward deepening sexual experience. Because attending to oscillating stimuli produces enhanced neural activity at corresponding frequencies (Andersen, Müller, & Hillyard, 2015; Mahajan, Davis, & Kim, 2014; Porcu, Keitel, & Müller, 2013), the rewarding nature of sexual attending increases the probability that entrainment is likely to occur. Preliminary evidence suggests that a bidirectional relationship between pleasure and entrainment exists for music perception (Trost et al., 2014; Trost & Vuilleumier, 2013); it is not unreasonable to suspect that even stronger feedback amplification may occur with sexual activity.

Although entrainment-related attention may be of a highly concentrated variety, it is also possible that more diffusely focused attention may produce maximal pleasure in some individuals. This may vary not only between people but also within the same person depending on the nature of the sexual activity. Whichever aspects of sexual experience are most rewarding, similar positive feedback is likely to occur with respect to patterns of attending that facilitate those experiences.

Finally, these positive feedback loops between pleasurable sensation and attention drive physiological arousal, which itself produces further pleasurable sensations, thus creating an additional positive feedback loop with the body (Janssen, 2011; Janssen, Everaerd, Spiering, & Janssen, 2000; Toates, 2009). The sensations of arousal are both pleasurable in-and-of-themselves and also allow for more effective sexual stimulation (e.g. via erection or lubrication). That is, the affordances (Gibson, 1977) and signal transduction properties of an aroused body are better suited for facilitating positive feedback amplification of pleasure, possibly via mechanisms of neural entrainment.

## The neurophenomenology of sexual trance

The present model of orgasm and sexual trance – and ecstatic experience more generally – is one in which rhythmic perception and action lead to entrainment, enhancing perception of entraining stimuli, thereby further enhancing entrainment, thus creating a positive feedback cycle of deepening sexual absorption. In this way, progression into deeper states of sexual enjoyment may lead to qualitatively different experiential dynamics. Specifically, some aspects of the sexual response cycle may not be adequately described solely in terms of increased arousal and pleasurable sensation (Georgiadis & Kringelbach, 2012; Masters, 1966). Rather, many of the qualities of sexual pleasure may specifically be driven by a synchrony-facilitated state of sensory absorption leading to trance.

Introspection, experimental psychology, and physics all suggest that brains have a limited ability to process information (Dowd, Kiyonaga, & Egner, 2015; Fusser et al., 2011; Kiyonaga & Egner, 2013Kiyonaga & Egner, 2014; von Helmholtz, 1847). Our conscious field of awareness has a limited capacity, with upper bounds to how much we can be aware of at any point in time. We partially overcome these limitations by biasing the degree to which different representations are active within conscious experience (i.e. attention) (Beck & Kastner, 2009; Smallwood, Brown, Baird, & Schooler, 2012). However, this process is not without tradeoffs. Enhanced processing of some representations can come at the expense of reduced cognitive resources being available for others. A common metaphor for attention is a spotlight (Müller, Malinowski, Gruber, & Hillyard, 2003), which when pointed in some directions is not pointed elsewhere. Enhancing arousal and synchrony can both be thought of as means of increasing the brightness, focus, or width of this spotlight, but not unboundedly.

With respect to body awareness, intensely focusing on immediate sensations—such as those produced by rhythmic stimulation – is likely to reduce the amount of mental capacity available for other things, such as self-narratives (Damasio, 2012). Extreme sensate focus has the potential to crowd-out ruminative processes (Kerr, Sacchet, Lazar, Moore, & Jones, 2013; Meston, Hull, Levin, & Sipski, 2004; Michalak, Hölz, & Teismann, 2011) and even prevent the recruitment of neural resources for other mental simulations (Barsalou, 2009; Pezzulo et al., 2011; Simmons et al., 2005), including the kinds of imaginings required for modeling the self in the past, future, or other counterfactual situations (Smallwood et al., 2012; Speth et al., 2016). Such an experience of sensate focusing and altered self-processing may be most appropriately referred to as a kind of trance state. If this trance occurs in the context of another individual who is similarly absorbed, then it could potentially contribute to feelings of connectedness along with the expansion of self-other boundaries (Hove & Risen, 2009; Wiltermuth & Heath, 2009).

Music and dance may be the only things that come close to sexual interaction in their power to entrain neural rhythms and produce sensory absorption and trance (Doelling & Poeppel, 2015). Indeed, this may be one of the primary reasons we sing and dance (Sievers, Polansky, Casey, & Wheatley, 2013; Trost & Vuilleumier, 2013). That is, the reasons we enjoy sexual experiences may overlap heavily with the reasons we enjoy musical experience, both in terms of proximate (i.e. neural entrainment and induction of trance-like states) and ultimate (i.e. mate choice and bonding) levels of causation (Tinbergen, 1963).

Some aspects of this model are likely unique to humans and their advanced capacity for high-level symbolic cognition (Tomasello, 2014). However, many (perhaps most) animals may be capable of experiencing sensual trance, perhaps even more readily than humans, especially if it is the case that many animals – who may have more limited narrative selves without the aid of syntactic language – are often in a state of sensory absorption (Fontani & Carli, 1997; Kurtz, 1975; Kurtz & Adler, 1973).

Even the psychological phenomenon of flow may be largely explainable in terms of entrainment-facilitated trance, because flow is often induced by directed attention towards rhythmic activities (Csíkszentmihályi, 1991; Tolstoy, 1914), or may involve rhythmic engagement with otherwise non-rhythmic tasks. Six factors have been identified as characterizing flow states (Csikszentmihalyi, 1997): 1) intensely focused concentration, 2) merged action and awareness, 3) loss of self-consciousness, 4) personal effectiveness, 5) alterations of subjective time, and 6) intrinsic reward. Most of these factors would be similarly apt for describing the kinds of peak experiences associated with sexual rhythms, and rhythm-induced trance-like states may be an important reason that sex is such an effective source of flow. Both sexual and non-sexual flow states may be rewarding because of enhanced engagement with pleasurable activities, allowing self-processes to be outcompeted for attentional resources, thus allowing for deeper pleasurable engagement.

Importantly, it should be noted that although sexual activity involving genital stimulation may be particularly conducive to sensory absorption, a trance model could also be appropriate for describing a variety of less-explicit sexual interactions, ranging from kissing to dancing, and perhaps even some kinds of intimate conversation.

## Evidence for sexual trance

This understanding of sexual experience as fundamentally related to trance or flow leads to many testable hypotheses. (For further hypotheses and proposed experiments, see Appendix.) If deactivation of frontal midline structures were observed during sexual interaction (something that would be difficult to measure in real time), then this might suggest disruption of self-related processing of the kind present in trance states (Heinzel et al., 2006; Nejad, Fossati, & Lemogne, 2013; Summerfield, Hassabis, & Maguire, 2009). Subsequent inclusion of another person into a self-concept could be evidenced if sexual interaction caused neural patterns in response to an erotic partner to become increasingly similar to patterns corresponding to self-related processing (Chavez & Heatherton, 2014). Similarly, sexual modification of body schemas to include another person – or other indicators of ‘mirroring’ (Lamm & Majdandžić, 2015) – could be tested via psychophysical experimentation (Blakeslee & Blakeslee, 2008; Cardinali et al., 2009).

Studies of sexual climax have already provided some (albeit inconsistent) evidence for deactivation of areas of cortex related to higher-order cognition and executive functions, including frontal and midline structures (Georgiadis & Kringelbach, 2012; Georgiadis, Kringelbach, & Pfaus, 2012; Holstege & Huynh, 2011), which were interpreted by investigators as reflecting the importance of a ‘loss of control’ in orgasmic functioning. However, considering the methodological challenges of studying sexual climax with neuroimaging, and given that this literature only has a few small-sample-size studies, caution is needed in interpreting either the presence or absence of findings. Furthermore, although numerous investigations have studied sexual arousal in response to passive viewing of stimuli (Stoléru, Fonteille, Cornélis, Joyal, & Moulier, 2012), these are not well suited for testing trance-related hypotheses, both because of participant immobility and lack of visceral stimulation, and also because the degree of sexual arousal experienced is likely to be low compared to naturalistic conditions.

## The neurophenomenology of orgasm

Much of orgasmic experience can probably be explained as an intensification of sexual pleasure and a deepening of the previously described altered states of consciousness. This likely contributes to explaining how people can be uncertain about whether or not they have actually had an orgasm (Darling & Davidson, 1986), or how an experience can be described as ‘orgasmic’ without actually involving sexual climax (Seecof & Tennant, 1986). However, it should also be noted that distinct emergent properties (Anderson, 1972) could be associated with different degrees of sexual absorption, perhaps with qualitatively different dynamics for different levels of trance.

In these ways, the neurophenomenology of orgasm is both similar to and continuous with other forms of sexual trance. However, unique aspects of orgasmic experience arise from major physiological and neuroendocrine changes accompanying sexual climax. Specifically, when evolutionarily conserved central pattern generators receive a sufficient amount of combined bottom-up and top-down stimulation (Johnson, 2006; Komisaruk & Whipple, 2011), multiple kinds of rhythmic smooth muscle contractions are triggered in the pelvic region (Bohlen, Held, & Sanderson, 1980; Bohlen, Held, Sanderson, & Ahlgren, 1982; Truitt & Coolen, 2002; van Netten, Georgiadis, Nieuwenburg, & Kortekaas, 2008). Furthermore, these central pattern generators may also send collaterals to both hypothalamic and brainstem nuclei that regulate neuromodulator, neuropeptide, and opioid levels (Pfaus et al., 2012; Young & Alexander, 2012). Alternatively, more indirect pathways might be involved, as would be the case if spontaneous muscle contractions themselves caused systemic neurochemical changes, perhaps via ascending inputs from stretch-receptor stimulation (Ferguson, 1941; Odent, 1987). Regardless of the specific causes of release of these neuromodulatory substances, the likely neural consequences are increased excitation and disinhibition of multiple neural systems, as well as enhanced plasticity in the most active neuronal networks (Camacho, Portillo, Quintero-Enríquez, & Paredes, 2009; He et al., 2015; Pan, Schmidt, Wickens, & Hyland, 2005). Moreover, to the extent that trance is associated with strongly synchronous neural activity, this may allow for the greatest degree of convergent inputs to hypothalamic and brainstem structures, thus causing the release of these hormones and neurochemicals to coincide with peak trance.

Orgasm has been likened to the rush of heroin injection (Chessick, 1960; Holstege et al., 2003), implying greatly elevated opioid concentrations. Stimulation of opioid receptors in the ventral pallidum is associated with liking (Berridge, Robinson, & Aldridge, 2009; Mitrovic & Napier, 1995), with varying effects in other brain locations (Mazei-Robison & Nestler, 2012).^4^ Although one might expect all excitation-promoting neurochemicals to be uniquely elevated at orgasm, this is not the case. Notably, although the initial stage of sexual climax may involve initially elevated levels of arousal-enhancing neuromodulators such as dopamine and norepinephrine (up to 195% more than baseline in rodents), these catecholamine elevations are not substantially greater than those observed during the pre-ejaculatory appetitive phase of sexual activity and even exhibit an immediate and drastic post-ejaculatory reduction (albeit still somewhat elevated relative to baseline) (Blackburn, Pfaus, & Phillips, 1992). It may be that the neuropsychopharmacology of sexual trance most closely mimics the effects of cocaine, with orgasm itself mimicking the addition of heroin to this pre-existing altered state (Seecof & Tennant, 1986). Climax is also associated with elevated oxytocin and vasopressin, both of which have been associated with analgesic-type effects (Baskerville & Douglas, 2010; González-Hernández, Rojas-Piloni, & Condés-Lara, 2014; Mogil et al., 2011), as well as enhanced social learning (Freeman & Young, 2016). In these ways, the intense pleasure of orgasm is produced by a complex (probably opioid-dominated) neurochemical cocktail being administered during a peak experience of sensual trance, thus creating an even deeper state of sexual ecstasy.

## Orgasm, seizures, and high-threshold reproductive systems

Komisaruk and Whipple have observed striking similarities between orgasm and seizures, pointing to several lines of evidence that ‘suggest a basic commonality between the two phenomena’ (Komisaruk & Whipple, 2011). First, they note some forms of epilepsy are capable of generating orgasm-like pre-ictal auras. Second, they describe seizures as characterized by abnormally large degrees of neural synchrony and go on to speculate that elevated synchrony also accompanies ‘the rhythmical and voluntary movement-generated timing of genital stimulation’. Finally, they observe that some aspects of orgasmic expression can be likened to the uncoordinated motor responses present during grand mal seizures and argue that widespread fMRI activations during orgasm suggest similar mass activations as observed in epilepsy.^5^

In light of case studies of epilepsy with orgasmic auras (Calleja, Carpizo, & Berciano, 1988; Fadul, Stommel, Dragnev, Eskey, & Dalmau, 2005; Janszky et al., 2004), and also considering the subjective qualities of orgasm more generally, it is possible that the processes involved in sexual climax may be similar to the dynamics of reflex seizures with absences. The causes of reflex seizures are variable, ranging from simple visual or auditory rhythmic inputs, to more complex and sometimes even conceptual triggers (Ferlazzo, Zifkin, Andermann, & Andermann, 2005; Koepp, Caciagli, Pressler, Lehnertz, & Beniczky, 2016; Xue & Ritaccio, 2006). In two particularly intriguing case studies, orgasmic seizures were triggered by tooth-brushing (Chuang, Lin, Lui, Chen, & Chang, 2004; Haytac, Aslan, Ozcelik, & Bozdemir, 2008). Notably, both sexual stimulation and tooth brushing involve rhythmic stimulation via high-bandwidth sensory channels. Similarly, in the case of epilepsy triggered by orgasm (Sengupta, Mahmoud, Tun, & Goulding, 2010), it is possible that the seizures are partially caused by extended periods of rhythmic sexual stimulation, or via elevated excitatory neural activity during orgasm itself, or both. Considering the importance of neural synchrony in the pathogenesis of seizures (Sobayo et al., 2012), these particular case studies are consistent with rhythmic entrainment as being central to sexual experience and orgasm.

For both sexual stimulation and reflex seizures, rhythmic inputs may help to entrain a synchronized functional network through which neural signals can more readily propagate (Liao et al., 2010; Netoff, Clewley, Arno, Keck, & White, 2004; Ponten, Bartolomei, & Stam, 2007). With respect to sexual stimulation, elevated synchrony could promote greater intensity of experience and perhaps paroxysmal events. With respect to seizures, elevated synchrony could promote conditions under which anomalous firing patterns are more likely to percolate across the brain and thus produce much of the pathophysiology of epilepsy. More specifically, if cascades of neural firing propagate simply because they are good at spreading, then these patterns may be able to disrupt functional neuronal signaling (Bartolomei & Naccache, 2011). Depending on where the dysregulated activity spreads, this disrupted information processing could interfere with the ability of the brain to model self and world, thus disrupting consciousness. These activation cascades, however, may be more self-limiting in orgasm and more dysregulated in epilepsy.

Notably, seizures with orgasmic auras may have originating foci in right inferolateral and temporal lobe structures (Aull-Watschinger, Pataraia, & Baumgartner, 2008; Janszky et al., 2002), which in other forms of epilepsy involve ecstatic states preceding lost consciousness (Picard & Craig, 2009; Picard & Kurth, 2014; Voskuil, 2013). In these ways, epilepsy-like dynamics may contribute to the phenomenology of orgasm as a ‘little death’. Or (but not mutually exclusively), sufficient explanation of disrupted consciousness may be found in the previously described positive-feedback intensification contributing to trance, or the potentially soporific effects of elevated opioids (Reinoso-Barbero & de Andrés, 1995).

The potential role of the temporal lobes in orgasm is further implicated by hippocampal theta rhythms, which build over the course of copulation in both male and female rats, with termination of sexual activity associated with an immediate breakdown of synchrony, as well as increased spindle activity (Kurtz, 1975; Kurtz & Adler, 1973). Theta rhythms are also heavily implicated in models of temporal lobe epilepsy (Arcaro et al., 2016; Broggini, Esteves, Romcy-Pereira, Leite, & Leão, 2016; Sedigh-Sarvestani et al., 2014), for which the hippocampus is a common locus of seizure initiation. Spindle activity is commonly interpreted as a marker of reactivation of hippocampal-based memories (Andrade et al., 2011; Kahn & Shohamy, 2013; Mednick et al., 2013). In theory, increased copulatory-phase theta activity could indicate the establishment of a network of enhanced pre-orgasmic functional connectivity, with subsequent spindle activity indicating the learning of partner characteristics from associated sexual stimulation. This speculative mechanism is one of many means by which orgasm could facilitate mate choice, by associating features from climax-inducing partners with the rewarding nature of both copulation and orgasmic experience (Pfaus, Kippin, & Centeno, 2001).

In noting parallels between orgasm and seizures, Komisaruk and Whipple (2011) also propose that widespread synchronous activity may act as a means of triggering ‘high-threshold systems, such as the system that controls ejaculation’. There are multiple evolutionarily sensible reasons for sexual climax not to be triggered too easily, such as avoiding wasteful energetic expenditures, missed mating opportunities, poorly chosen mating partners, potentially dangerous situations from poorly timed distractions, or failing to provide adequately reinforcing stimulation to a desired partner. In the following sections, I will describe potential factors influencing orgasms as variable-threshold systems, considering both proximate mechanisms and evolutionary selective pressures.

## Surpassing thresholds: variable impacts of rhythms and variable intensity orgasms

The discussion above has focused on the rhythmic nature of sexual stimulation as being central to erotic experience, suggesting that sexual arousal may be fruitfully understood as a kind of trance state, facilitated by positive feedback between entrainment and pleasurable sensory absorption. Particularly robust neural activity enabled by entrainment-enhanced synchrony may constitute a means of surpassing the high thresholds of stimulation required for orgasm and associated neuroplastic processes.^6^

However, similarly to seizure thresholds (Maguire & Salpekar, 2013), orgasm thresholds are not static properties of brains that are neatly determined by rhythmic stimulation patterns. Rather, induced synchronous activity is a function of multiple factors, including the firing thresholds of individual neurons, with overall excitability tending to be increased with physiological arousal (Maguire & Salpekar, 2013). Indeed, it is notable that different neuropharmacological factors impact thresholds for seizures and sexual climax in similar ways, likely reflecting overall propensity for activation cascades to spread through neuronal networks: thresholds increase with elevated GABA receptor stimulation (Brannon & Rolland, 2000; Calabrò, De Luca, Pollicino, & Bramanti, 2013; Clark & Elliott, 1999), but decrease with elevated glutamate receptor stimulation (Bishop, Chae, Patel, Moline, & Ellingrod, 2012; Crino et al., 2002), dopamine agonists (Andersen & Tufik, 2005; Komisaruk, Beyer-Flores, & Whipple, 2008; Purcell et al., 2013), and dopamine/norepinephrine reuptake inhibitors (Abdel-Hamid & Saleh, 2011; Kim & Steinhart, 2010; Labbate, 1998; Modell, May, & Katholi, 2000).^7^

Theoretically, modulation of these kinds of general excitatory or inhibitory signaling pathways could provide a mechanistic explanation for the influence of hormones on orgasmic variability across the menstrual phase (Reddy, 2012; Udry & Morris, 1968). It could also explain how the perceived significance of a sexual event or partner (Gallup, Ampel, Wedberg, & Pogosjan, 2014) could influence sexual responsiveness and motivation via enhanced excitatory signaling or elevated salience-related neuromodulators such as dopamine or norepinephrine (Blackburn et al., 1992).

With respect to sexual stimulation and orgasm, degree of entrainment and overall neuronal excitation are likely to be heavily modulated by factors such as degree of attending or arousal. Two sexual acts can be identical in their motoric properties, yet be experienced very differently based on partner or situational characteristics (Chivers & Timmers, 2012; Rupp et al., 2009). In this way, although particular external rhythms may be more or less conducive to sexual enjoyment, they are neither sufficient for explaining the degree to which internal rhythms are entrained, nor sufficient for explaining the likelihood or intensity of orgasms.

This kind of multidetermination for orgasmic responding (e.g. based on both partner rhythmic ability and perceived partner salience) may be adaptive for species with sophisticated cognitive abilities, because it allows greater flexibility in releasing the powerfully rewarding mechanisms underlying sexual trance and orgasm. More specifically, although rhythmic capacity may be an honest indicator of organismic fitness (Broek & Todd, 2009; Hugill et al., 2010; Neave et al., 2010; Röder et al., 2015), it may be evolutionarily adaptive to consider (or place even greater emphasis on) additional criteria in deciding whether to trigger high-threshold reproductive mechanisms. This may be because organisms with complex nervous systems may also encounter (or construct) complex and dynamic niches within which fitness criteria change rapidly. Or, it may be the case that biased sensitivities to some fitness-indicating features may be too complex to pre-program into the nervous system (e.g. predictors of the probability of having future resources in a particular culture). And so, flexible conditions for orgasm may have allowed natural selection to leverage the power of general-purpose learning mechanisms for flexibly adaptive mate choice, rather than solely relying on the evolution (and evolvability) of specific cognitive adaptations.

Specifically, as previously discussed, the high-threshold system of orgasm causes the release of multiple neuromodulators, which act as meta-plasticity factors to enhance learning of preferred partner characteristics, and thus influence mate choice and bonding. The result of this elevated plasticity is likely enhanced learning of the stimulus features associated with the sexual partner(s) that contribute to sexual climax (Brannon & Rolland, 2000; Calabrò et al., 2013; Clark & Elliott, 1999). Given that orgasm is one of the greatest pleasures available to an organism (Pfaus et al., 2012; Tenk et al., 2009; Toates, 2014; Wunsch, 2010), these stimulus consummatory features will be linked to high incentive value (via classical conditioning), and associated behaviors will be powerfully reinforced (via operant conditioning). In both males and females, these conditioned associations would have the effect of increasing the frequency of mating with particular individuals and thus increasing the probability of reproduction with those sexual partners. Furthermore, the flexible conditions for orgasm release mean that mate choice and bonding will be driven by both honest indicators of fitness and criteria that are significant to particular organisms within particular contexts.

Additionally, it should be noted that both orgasms and seizures vary not only in the frequency of their occurrence but also in their intensity (King, Belsky, Mah, & Binik, 2010; Leeners et al., 2013). Although surpassing a climactic threshold may be a particularly significant experiential event, it is important to keep in mind that sexual acts and orgasms can be qualitatively different and that differences in enjoyment and intensity can modulate the degree of conditioning. In men, these differences may influence ejaculate characteristics in ways that make a given fertilization more likely, possibly even at the expense of near-future fertilization attempts with other potential mates (Joseph, Sharma, Agarwal, & Sirot, 2015; Parker & Pizzari, 2010). In women, it has also been suggested that orgasms could influence the probability of conception (Pavlicev & Wagner, 2016; Puts et al., 2012), although this view is controversial (Levin, 2011); theoretically, more intense or multiple orgasms may have even greater influences on conception probabilities.

Importantly, the same multicausal conditions for evaluation of sexual partners are not only contingent upon orgasm having occurred but also take place over the entirety of sexual interaction, as well as before initiating sexual activity. As previously discussed, it may be the case that entrainment and trance may play a role in evaluating partner desirability during all stages of engagement with potential mates (e.g. via speech, dancing, kissing, and foreplay).

## The evolutionary bases of sex differences in orgasm

In considering the evolutionary bases of sex differences in orgasm, it is important to remember that males and females of many species confront different challenges and opportunities with respect to reproductive success (Buss & Schmitt, 1993). Specifically, human females have large obligate investments for successful reproduction, in that in order for offspring to survive women must invest approximately 9 months of gestation time, along with the associated risks of childbirth.^8^ Human males, in contrast, may be able to successfully reproduce with only a single sexual encounter. The lower- and upper-bound estimates for expected offspring also differ. Throughout history, most human females would be able to successfully achieve occasional fertilization, but could only produce as many offspring as their gestation times and lifespan would permit. Conversely, the variability in reproductive success among human males was relatively vast, with it not uncommon for some males to leave no surviving offspring, but for other males to produce many descendants (Karmin et al., 2015). Although both men and women possess evolutionary incentives for novelty preferences via genetic diversification – and perhaps other reasons as well (e.g. alliance facilitation) – it is undeniable that there were substantial differences in the upside and downside risks and opportunities for sexual interaction.

With these evolutionary considerations in mind, it should come as no surprise that many men achieve orgasm both reliably and (relative to women) indiscriminately, and it should be similarly unsurprising that many women have a large number of contingent factors that influence the frequency and quality of their orgasms (Gallup et al., 2014; Garcia, Lloyd, Wallen, & Fisher, 2014; Meston et al., 2004; Meston, Levin, Sipski, Hull, & Heiman, 2004). More specifically, the intense pleasure of orgasm is likely to influence the degree of incentive motivation for sex (Georgiadis et al., 2012; Toates, 2009). On average, the ability to have more frequent orgasms with a broader variety of partners should make men less discriminating in their choice of mates. On average, a more restricted ability for orgasm, which may only occur with a more selective variety of partners, should make women more discriminating in their choice of mates. Mechanistically, this kind of adaptation for differential mate choice thresholds may have been achieved by higher thresholds for orgasmic responding having evolved in women, relative to men.

Notably, although women may have higher thresholds for climax, they are also more likely to be capable of multiple orgasms (Darling, Davidson, & Jennings, 1991; Masters, 1966). Although this might seem inconsistent with increased partner discriminability, multi-orgasms could potentially enhance discrimination by increasing the discrepancies between more and less rewarding sexual experiences.

However, it should be noted that orgasmic response varies greatly between (and within) individuals, and in particular, women (Garcia et al., 2014; Hoon & Hoon, 1978). Thus, rather than expecting a singular and universal female-typical or male-typical orgasmic response profile, we should not be surprised by substantial variability within each gender, since an individual’s orgasmic functioning may reflect idiosyncratic developmental events, and possibly also particular evolutionary strategies (Wlodarski & Dunbar, 2015).

This is not to say that male orgasm – and bonding, given sufficient kindling of incentive motivation (Pfaus et al., 2012) – is homogenous and insensitive to context; this would be surprising in a species in which both sexes typically provide substantial levels of parental investment (Buss & Schmitt, 1993). More resources invested in offspring means larger associated opportunity costs, and so heavily investing male and female humans both require more stringent tests – relative to less-investing species (Pianka, 1970) – to ensure favorable returns on investment. These greater resource demands may be part of the reason that humans are relatively exceptional in the elaborateness of their sexual interactions, with rhythmic virtuosity and sensitivity being important for sexual prowess on the part of both males and females.

The above account is not meant to imply that every aspect of orgasmic expression directly contributes to reproductive success. Animals as intelligent and behaviorally flexible as humans have found numerous ways to deploy rhythmic pleasure for personal enjoyment and social desires (Merker, Morley, & Zuidema, 2015; Meston & Buss, 2009). For example, individuals of the same sex may use orgasms to help establish and maintain relationships that do not contribute to reproduction. However, it may also be the case that some bisexual and homosocial bonding may reflect a history of adaptation in facilitating social alliances (Fleischman, Fessler, & Cholakians, 2014; Sommer & Vasey, 2006).

## Conclusion

What is orgasm? This article has attempted to provide a multilevel evolutionary explanation involving enabling conditions, plausible neurophysiological mechanisms, and experiential as well as behavioral impacts of entrainment through rhythmic sexual activity. Much work remains to be done in order to adequately characterize this profound and fundamental experience. Future researchers may wish to contemplate the different evolutionary life history strategies that could be enabled by adjusting parameters for orgasmic responding in males and females, as these are likely to be continuous with sexual incentive motivation and capacity for bonding to one or more sexual partners on various timescales. In sex – as in so many other aspects of life – timing is everything.

## Footnotes

1 The model predicts that cases of purely psychogenic orgasm will often—but not necessarily always—be accompanied by some combination of a) imagined rhythmic sexual activities (Cichy, Heinzle, & Haynes, 2012; Simmons, Martin, & Barsalou, 2005; Slotnick, Thompson, & Kosslyn, 2005) and b) self-stimulation via rhythmic contractions of muscles located near regions of erogenous sensitivity.

2 In a similar vein, Dawkins (2006) intriguingly suggested that absence of a baculum may have allowed erectile stability to serve a similar fitness-signaling function in humans. In a different domain, not dissimilar proximate and ultimate causes may have also shaped responses to infant suckling (Tinbergen, 1963; Wang, Negoro, & Honda, 1996), where rhythmic stimulation of maternal breast tissue is necessary for promoting the milk let-down reflex, and where the ability to produce such stimulation may be an honest indicator of fitness on the part of infants, which in non-human mammals would have the adaptive payoff of terminating investment in non-viable offspring.

3 More complex structures and processes may also implicate synchrony as important for propagating neural signals. Some examples include synchronous generation of local field potentials (Anastassiou, Perin, Markram, & Koch, 2011; Buzsáki, Anastassiou, & Koch, 2012; Qiu, Shivacharan, Zhang, & Durand, 2015), synchronous convergence into network hubs of the brain (Damasio, 2012; Modha & Singh, 2010; van den Heuvel & Sporns, 2011), as well as specialized coordination-structures such as the claustrum and reticular nucleus of the thalamus (Crick & Koch, 2005; Landisman et al., 2002; Smythies, Edelstein, & Ramachandran, 2012).

4 Stimulation of kappa opioid receptors may be partially responsible for the dysphoric symptoms experienced by some individuals during and/or after climax (Kivell et al., 2014; Schweitzer, O’Brien, & Burri, 2015).

5 The parallels between these observations and ideas described in this article are striking, since the hypothesis of sexual entrainment was developed prior to encountering this publication by Komisaruk and Whipple (personal communication sent to Sexnet email listserv, 2011). This theoretical convergence could potentially be considered to be a form of support for the idea that rhythms are central for understanding orgasm.

6 Robustly driven neural activity likely also underlies the maladaptive plasticity contributing to epilepsies that tend to progress in severity (Sutula, 2004).

7 However, these literatures do not show straightforward correspondences between neuron-level excitation and orgasm (or epilepsy), with discrepant findings potentially being caused by emergent properties at the level of physiological networks (e.g. disinhibition and nonlinear dynamics), impacts on psychological and physical processes (e.g. difficulties performing sexually or focusing on sexual sensations under some altered states), or confounds between acute and chronic drug effects (e.g. physiological adaptation or comorbid psychopathology).

8 Extensive nursing and post-nursing care is likely required for successful reproduction, although this load may have been partially reduced if it were common for there to be closely bonded extended families or community co-parenting arrangements in environments of evolutionary adaptation (Royle, Russell, & Wilson, 2014).

## Appendix A: Methods for testing sexual entrainment

Neural entrainment may or may not be directly involved in the release of brainstem and spinal fixed action patterns for sexual climax. Studies of biological and robotic systems have demonstrated that qualitatively different dynamics can be produced in pattern-generating neural systems merely by changing overall levels of control energy (El Manira & Grillner, 2008; Ijspeert, Crespi, Ryczko, & Cabelguen, 2007). It could be the case that rhythmic stimulation at particular frequencies is maximally effective at causing these mode shifts (McClellan & Jang, 1993; McClellan & Sigvardt, 1988), but it is also possible that neural entrainment may not play a significant role.

Spontaneous 8–13 Hz rectal contractions are a reliable signal of orgasm taking place in humans, and presumably some non-human animals (van Netten et al., 2008). It is possible that this is merely the spontaneous driving mode of central pattern generators. But notably, this is approximately proportional to the rate of high-frequency stimulation that is common for sexual activity (Levin, 2006).^9^ However, it may also be the case that central pattern generators are most effectively triggered to enter particular bursting modes when inputs occur at those frequencies, thus implicating entrainment-type processes. It is even possible that these rectal contractions may also be related to the frequency of synchronous activity in upstream dynamics, because climax involves a combination of bottom-up and top-down inputs.

The hypothetical brainstem mechanisms discussed above would be investigated most effectively using invasive methods in awake-behaving animals. Cortical processes would also be beneficially investigated in these ways, but some of these dynamics might be particular to humans, who require the use of non-invasive methods.

Electroencephalography or magnetoencephalography could be used to test whether sexual activity results in increased coherence between sensors. If measurements of increasingly distant nodes become increasingly correlated over the course of sexual stimulation, and if the frequency of correlated activity is related to stimulation frequency, then this would represent strong evidence that neural entrainment takes place during sexual activity. Conversely, the absence of such evidence – with an adequately powered study – would falsify the model.

Functional magnetic resonance imaging or near-infrared spectroscopy could provide indirect evidence for entrainment if sexual stimulation produced increasing degrees of functional connectivity between increasingly distant brain areas. However, these methods would be unsuitable for falsification, because their poor temporal resolution would make it difficult to interpret null findings.

Another experimental approach would involve pulsing focal areas of cortex with transcranial magnetic stimulation (or perhaps transcranial direct current stimulation) and then measuring signal propagation distance (Bortoletto, Veniero, Thut, & Miniussi, 2015). Theoretically, propagation distance would increase over the time-course of sexual stimulation – potentially also reflected by increased small-world network properties (Sänger et al., 2012) – and also correlate with degree of sexual absorption.

Weak-to-moderate support for entrainment could be obtained if music, dance, or some other rhythmic practice enhanced sexual and orgasmic functioning. Stronger support would be obtained if adaptive neural entrainment devices were shown to be effective interventions for either clinical practice or personal enhancement (Santostasi et al., 2016).

Finally, substantial (but not insurmountable) technical challenges are faced in attempting to test for entrainment *between* nervous systems (Acquadro, Congedo, & De Riddeer, 2016; Sänger, Müller, & Lindenberger, 2013), including the possibilities of insufficiently corrected neuromuscular and motion artifacts in electromagnetic signals, as well as additional ways in which spurious associations might be observed (Burgess, 2013).
